# Transformer-Based Foundation Learning for Robust and Data-Efficient Skin Disease Imaging

**DOI:** 10.3390/diagnostics16030440

**Published:** 2026-02-01

**Authors:** Inzamam Mashood Nasir, Hend Alshaya, Sara Tehsin, Wided Bouchelligua

**Affiliations:** 1 Human-Environment-Technology (HET) Systems Centre, Mykolas Romeris University, 08303 Vilnius, Lithuania; 2 Applied College, Imam Mohammad Ibn Saud Islamic University (IMSIU), Riyadh 11432, Saudi Arabia; hialshaya@imamu.edu.sa; 3 Faculty of Informatics, Kaunas University of Technology, 51368 Kaunas, Lithuania; sara.tehsin@ktu.edu

**Keywords:** dermoscopic lesion imaging, dermoscopy, foundation model, vision transformer, self-supervised learning, cross-dataset generalization, medical image analysis

## Abstract

**Background/Objectives:** Accurate and reliable automated dermoscopic lesion classification remains challenging. This is due to pronounced dataset bias, limited expert-annotated data, and poor cross-dataset generalization of conventional supervised deep learning models. In clinical dermatology, these limitations restrict the deployment of data-driven diagnostic systems across diverse acquisition settings and patient populations. **Methods:** Motivated by these challenges, this study proposes a transformer-based, dermatology-specific foundation model. The model learns transferable visual representations from large collections of unlabeled dermoscopic images via self-supervised pretraining. It integrates large-scale dermatology-oriented self-supervised learning with a hierarchical vision transformer backbone. This enables effective capture of both fine-grained lesion textures and global morphological patterns. The evaluation is conducted across three publicly available dermoscopic datasets: ISIC 2018, HAM10000, and PH2. The study assesses in-dataset, cross-dataset, limited-label, ablation, and computational-efficiency settings. **Results:** The proposed approach achieves in-dataset classification accuracies of 94.87%, 97.32%, and 98.17% on ISIC 2018, HAM10000, and PH2, respectively. It outperforms strong transformer and hybrid baselines. Cross-dataset transfer experiments show consistent performance gains of 3.5–5.8% over supervised counterparts. This indicates improved robustness to domain shift. Furthermore, when fine-tuned with only 10% of the labeled training data, the model achieves performance comparable to fully supervised baselines. **Conclusions:** This highlights strong data efficiency. These results demonstrate that dermatology-specific foundation learning offers a principled and practical solution for robust dermoscopic lesion classification under realistic clinical constraints.

## 1. Introduction

Skin cancer and other inflammatory or neoplastic dermatoses represent a growing global health burden, with incidence rising across many regions due to aging populations, lifestyle factors, and increased ultraviolet exposure. Accurate and timely diagnosis is crucial for reducing morbidity and mortality, particularly for melanoma, where early-stage detection strongly correlates with survival. Dermoscopic imaging has become a standard tool for enhancing visual assessment, yet performance remains constrained by inter-observer variability and limited access to expert dermatological care in many settings. Recent reviews highlight how artificial intelligence (AI) and deep learning are increasingly integrated into dermatology workflows to support screening, triage, and decision-making, but also emphasize that robust performance depends on large, diverse image repositories and careful handling of dataset shift and demographic imbalance [1,2,3]. In particular, systematic analyses of skin cancer image datasets show that non-representative cohorts and heterogeneous acquisition protocols can adversely affect downstream model generalizability, underscoring the need for algorithmic designs that are resilient to real-world distribution shifts [1].

Early generations of AI systems for dermoscopic analysis have been dominated by convolutional neural networks (CNNs) and their hybrids with attention modules, which achieve strong performance in controlled benchmarks but face structural limitations. Recent research has introduced new, more complex variations of CNN’s or hybrid (deep feature extraction, ensemble learning framework, attention-based residual networks) CNNs that are used for multi-class classification of skin cancer by way of dermoscopy [4,5,6,7,8]. These methods have shown how improving local feature encoders and incorporating better fusion methods results in the increased sensitivity to fine patterns of pigmentation and lesion margins, though these models continue to depend upon fixed local receptive fields and limited ability to take into account longer-range spatial relationships, which are important for discriminating between visually similar lesion classes. The effectiveness of some of the CNN models trained using a single dataset is decreased when assessed with external datasets due to the impact of domain shift created by differences in the imaging devices, settings used when capturing the images, and the characteristics of the patient population [1,6]. This limitation makes it hard to implement these models in practice, as they need to be effective when tested across a range of institutions, with people of different skin colors, and under varying prevalence rates.

Motivated by these challenges, vision transformers and related attention-based architectures have been introduced into dermoscopic image analysis to better model global context and complex lesion morphology. Several recent studies report that transformer backbones, including vanilla Vision transformers (ViT) and hierarchical variants such as Swin Transformer, can outperform conventional CNNs in skin lesion classification and combined segmentation–classification pipelines, especially for melanoma screening scenarios where subtle global structure and multi-scale context are important [9,10,11]. Hybrid designs that integrate transformer blocks with convolutional stages or other advanced encoders further enhance performance and robustness, for example, through early-stage CNN feature extraction followed by transformer-based global modeling and fusion [7,12,13]. Despite these gains, most transformer-based dermatology models are still trained in a fully supervised manner on limited datasets and are typically evaluated in closed-set or single-dataset configurations, leaving questions about cross-dataset generalization and scalability under limited annotation budgets largely unresolved.

In parallel with these architectural advances, the broader medical imaging community has begun to explore foundation models trained on large-scale heterogeneous data, with dermatology emerging as a prominent early application domain. Recent studies discuss the promises and pitfalls of foundation models in dermatology, highlighting their potential for improved accuracy, fairness, and data efficiency, while also cautioning about biases, transparency, and deployment challenges [14,15]. Large multimodal dermatology foundation models have been introduced that are pretrained on millions of clinical photographs and dermoscopic images, often leveraging self-supervised or contrastive objectives and evaluated across diverse downstream tasks ranging from screening to risk stratification and change monitoring [16,17]. Related efforts align powerful vision backbones with large language models to build interactive diagnostic assistants, such as SkinGPT-4, which combine image understanding and natural language reasoning for dermatological consultation [18]. At the same time, recent work has begun to systematically assess the trustworthiness and subgroup performance of such foundation architectures, for example, by evaluating segmentation-oriented foundation models on dermoscopic datasets such as HAM10000 and examining performance variations across diagnoses, demographics, and anatomical sites [19]. These developments indicate a clear shift from task-specific networks to general-purpose dermatology foundation models, yet most existing systems are either multimodal clinical tools or focus on segmentation and risk prediction rather than serving as reusable, image-centric backbones tailored specifically for dermoscopic classification across datasets.

Self-supervised learning (SSL) has emerged as a key enabling technology for scaling dermatology models beyond the limitations of labeled data, particularly for dermoscopic imaging. Recent approaches have shown that SSL can effectively pretrain encoders on large collections of unlabeled dermoscopic and clinical photographs, substantially improving downstream melanoma and dermoscopic lesion classification performance in low-label regimes [20,21,22]. Moreover, multimodal SSL strategies that jointly leverage dermoscopic and clinical views or auxiliary lesion attributes have been proposed to capture complementary information and reduce reliance on extensive manual annotation [21]. Building on these ideas, newer frameworks combine SSL with active or unsupervised domain adaptation to explicitly improve cross-dataset generalization, demonstrating that SSL-pretrained models followed by adaptation procedures can outperform purely supervised baselines on multiple target domains with varying degrees of shift [23]. Despite this progress, there remains a limited number of works that systematically develop and evaluate a dermatology-specific transformer foundation model trained in a self-supervised manner on dermoscopic imagery and rigorously benchmarked under both in-dataset and cross-dataset conditions.

While recent advancements in deep learning techniques have improved the ability to analyze dermoscopic images, many significant challenges limit their deployment in the real world. Most current solutions use Fully Supervised Paradigms for training, utilizing a single dataset and being highly sensitive to dataset bias, annotation scarcity, and domain shift from different devices and clinical environments. The challenge in dermatology is that expert annotators are expensive, and the distribution of images across institutions and populations is diverse. All of this makes it difficult to create robust, generalizable models.

The main contributions of this research study can be summarized as follows:

- A Transformer-based foundation model specifically developed for dermoscopic image analysis that utilizes dermatology-oriented self-supervised pretraining and hierarchical token fusion to capture multi-scale lesion features;
- A comprehensive and multidimensional experimental validation of the performance of the proposed approach on three public dermoscopic image datasets, including in-dataset and cross-dataset performance and robustness to limited label training; and
- A detailed ablation and efficiency analysis enabling readers to understand how self-supervised initialization and architectural design choices improve diagnostic performance and speed of convergence.

Together, these contributions yield a cohesive, practical framework for classifying dermoscopic lesions under realistic clinical constraints.

The remainder of this paper is organized as follows. Section 2 reviews recent related work in dermoscopic lesion analysis and dermatological foundation modeling. Section 3 describes the proposed Transformer-Based Foundation Model, including the hierarchical architecture and self-supervised pretraining strategy. Section 4 presents the experimental results, covering in-dataset performance, cross-dataset generalization, limited-data evaluation, ablation analysis, and computational efficiency studies. Finally, Section 5 concludes the paper and outlines potential directions for future research.

## 2. Literature Review

Recent developments in dermatological image analysis have demonstrated a clear transition from conventional convolutional neural networks toward attention-based and hybrid deep learning architectures. The introduction of transformer models has enabled more effective modeling of long-range dependencies and lesion-level contextual relationships that are not easily captured by localized convolution operators. On the ISIC 2018 benchmark, DermViT proposed diagnosis-guided attention embedding to strengthen category separability in complex lesion phenotypes [24], while Swin-Transformer pipelines utilized shifted-window multi-scale attention mechanisms to enhance spatial coherence in dermoscopic analysis [25]. Segmentation-classification hybrid pipelines further refined diagnostic discrimination by providing lesion localization priors to subsequent transformer classification blocks [9]. The further development of vision transformers using segmentation and classification models [26], along with improved boundary awareness and color/texture fusion from deep learning CNN-Transformer ensembles [27], will yield more accurate results, even in areas with high class imbalance.

Various research methods have been used to improve the reliability of HAM10000 datasets. They have taken three approaches: ensemble strategy, multimodal fusion, and architecture optimization. More recently, they have started to use models in real time through deployment. Using deep CNNs in conjunction with ensemble methods yields a set of Gaussian predictive distributions for each backbone network. The resulting collections of predictive distributions are used to stabilize classifications of HAM10000 datasets by eliminating class imbalance [5]. Furthermore, multimodal deep learning models that combine patient metadata and dermoscopic images were found to produce greater inter-class separation than single-stream image networks [28]. Also, the use of YOLOv8-based dermatology pipelines enabled researchers to implement detection–classification architectures based on lightweight algorithms, enabling near-real-time lesion evaluation [29]. A systematic review of optimizing hybrid CNN–Transformer models and hyperparameter tuning to improve classification performance has been published [30]. In addition, two-stage learning, consisting of coarse and refined classifiers, has been found to enhance decision consistency across all lesion subcategories [31]. These four methodologies, each employing different research themes, suggest that when optimizing the performance of the HAM10000 datasets, researchers should consider how multiple modalities, model diversity, and efficient training techniques interact.

The PH2 dataset is a recognized standard for Comparative Small-scale Transfer Learning Benchmarking; as such, this dataset illustrates numerous novel approaches to Low Data Regime Optimization and Attention-Augmented Classification. LesionNet provided superior baseline results using residual convolutional networks that have been optimized for the purpose of performing texture discrimination [32]; and furthermore, the integration of self-attention transformer units allowed for extended capability to aggregate contextual pattern information while still providing the same efficiencies previously noted in utilizing Convolutional Neural Networks (CNNs) [33]. Dual-branch networks that incorporate simultaneous segmentation and classification objectives achieve improved melanoma discrimination by enforcing boundary-aware representation learning [34]. Further works employed attention-guided CNN frameworks to emphasize lesion-specific saliency masking to improve decision reliability [35], while compact transformer-CNN fusion models aimed to improve generalization on small datasets via cross-feature reinforcement strategies [36]. These PH2 benchmarks underscore the value of attention fusion and task coupling in limited-annotation scenarios. A consolidated overview of representative baseline models discussed throughout this section is reported in Table 1.

## 3. Proposed Methodology

**Figure 1:**
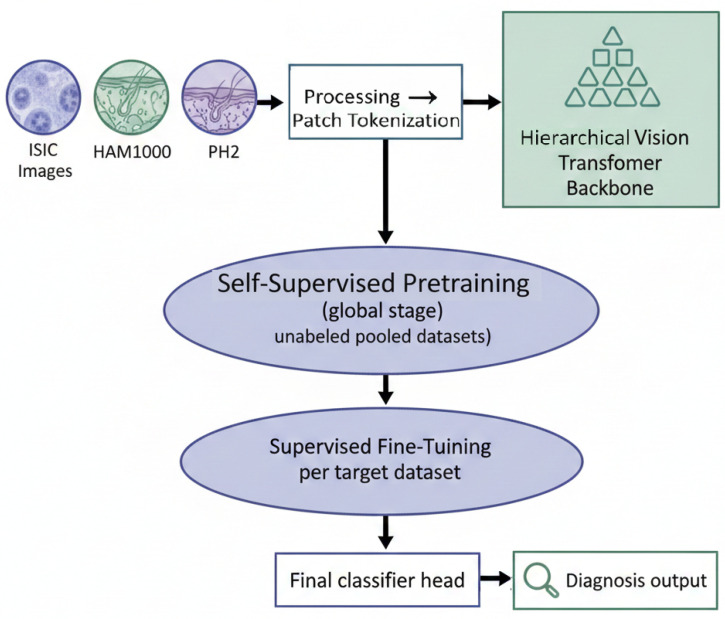
Overall framework of the proposed dermoscopic Transformer foundation model.

In this section, we present the proposed transformer-based foundation model for dermoscopic lesion imaging. The overall pipeline is designed to leverage large-scale self-supervised pretraining on heterogeneous dermoscopic image collections, followed by task-specific supervised fine-tuning for multi-class dermoscopic lesion classification. The model is built on a hierarchical vision transformer backbone that operates on patch-level representations of dermoscopic images and learns rich global contextual features. The foundation model is first trained data-drivenly, without labels, to capture generic dermoscopic structures and textures, and is subsequently adapted to downstream diagnostic tasks across different dataset configurations. The complete pipeline of the proposed Transformer foundation model, from multi-dataset input to diagnosis output, is summarized in Figure 1.

### 3.1. Formal Definition of Core Model Components

Let $X\in R^{H\times W\times 3}$ denote an input dermoscopic image with height *H*, width *W*, and three color channels. The image is partitioned into non-overlapping P×P patches, yielding $N=HW/P^{2}$ patches. Each patch is flattened and linearly projected into a *D*-dimensional embedding space using a learnable projection matrix, yielding the input token sequence.

(1) $$Z=\left[z_{1},z_{2},\ldots ,z_{N}\right],\;z_{i}\in R^{D}.$$

Positional embeddings are added to preserve spatial information, forming the transformer input representation. Given the embedded token sequence *Z*, multi-head self-attention is computed by projecting tokens into query, key, and value representations:

(2) $$Q=ZW_{Q},\;K=ZW_{K},\;V=ZW_{V},$$

where $W_{Q}$, $W_{K}$, and $W_{V}$ are learnable parameter matrices. The attention operation is defined as

(3) $$\mathrm{Attention}\left(Q,K,V\right)=\mathrm{softmax}\;\left(\frac{QK^{⊤}}{\sqrt{d}}\right)V,$$

with *d* denoting the dimensionality of each attention head. Multi-head attention aggregates information from multiple representation subspaces and is followed by residual connections and feed-forward transformations to produce refined token embeddings. After the final transformer stage, a global feature representation $f\in R^{D_{f}}$ is obtained via token aggregation. This representation is mapped to class logits using a linear classification head:

(4) $$o=W_{c}f+b_{c},$$

where $W_{c}\in R^{C\times D_{f}}$ and $b_{c}\in R^{C}$ denote the classification weights and bias, and *C* is the number of disease categories. The final class probabilities are obtained through the softmax function,

(5) $$p\left(y=c|X\right)=\frac{\exp(o_{c})}{\sum_{k=1}^{C}\exp\left(o_{k}\right)}.$$

### 3.2. Transformer-Based Foundation Model

Let $X\in R^{H\times W\times 3}$ denote a dermoscopic image with spatial resolution H×W and three color channels corresponding to red, green, and blue intensities. The proposed foundation model begins by applying min–max normalization, followed by geometric resizing, so that all input images are mapped to a unified spatial resolution compatible with batch-based training and hierarchical attention modeling. After normalization, each processed image is partitioned into a set of non-overlapping square patches of uniform spatial size P×P, where *P* denotes the predefined patch length measured in pixels. This partitioning process transforms the two-dimensional image into a structured token representation by flattening each patch into a vector of dimension $3P^{2}$. Let $N=\frac{HW}{P^{2}}$ represent the total number of extracted patches. Each flattened patch vector is linearly projected onto a latent feature space of dimension *D* using a trainable affine transformation whose parameters consist of a projection matrix $W_{p}\in R^{3P^{2}\times D}$ and a bias vector $b_{p}\in R^{D}$. The embedded token sequence produced by this operation is thus arranged into a patch token matrix $Z_{0}\in R^{N\times D}$, where each row $z_{i}$ corresponds to the projected feature embedding of the *i*-th patch.

(6) $$Z_{0}={\left[z_{1},z_{2},\ldots ,z_{N}\right]}^{T}$$

The representation of each token vector $z_{i}\in R^{D}$ serves as a representation for its particular localized anatomical region(s) from the original dermoscopic picture. This maintains all pertinent information for dermatologic diagnosis, including, but not limited to, the texture, colour, pigment distribution, and spatial arrangement of the anatomical feature. As with most transformer models, however, the architecture cannot use token spatial ordering on its own. Therefore, additional information about the location of this anatomical feature on the original grid must be provided as part of the token embeddings. To provide such information, a matrix of positional embeddings $E_{pos}\in R^{N\times D}$ has been created, where the *i*-th row $e_{i}$ denotes the spatial encoding associated with the relative position of patch *i* in the initial dermoscopic grid. The sequence fed into the transformer backbone is created by combining the patch embeddings via element-wise addition of the positional embedding associated with each token. Thus, each token includes not only information about the characteristics and appearance of that patch, but also its original location on the original dermatosis grid before it passes through a self-attention network.

(7) $$X_{0}=Z_{0}+E_{pos}$$

The sequence of tokens, $X_{0}\in R^{N\times D}$, serves as input to a hierarchical transformer backbone comprising *L* stacked attention stages, each modeling long-range dependencies and gradually integrating information across all lesion areas within its context. The token feature for each attention stage l∈{1,2,…,L} is updated by a series of attention transformations and feedforward transformations, which ultimately enable the network to convert local features into holistic features that inform the irregularities of lesion shapes and color distributions, as well as the variations at the boundaries of lesions. The backbone parameters during pretraining of the foundation model are updated by optimizing them with self-supervised objectives directly applied to $X_{0}$ and its transformations, thereby eliminating the need for class-level labels. The self-supervised objectives guide the network to reconstruct masked tokens and/or to correlate multiple-view representations of the same dermoscopic image, thereby enabling it to generate a global representation of each dermoscopic image based on its inherent image structure. Upon convergence of the pretraining phase, the parameters of the trained backbone are transferred to the supervised learning environments by appending a lightweight, task-specific classification head (a weight matrix, $W_{c}\in R^{C\times D}$, and a bias vector, $b_{c}\in R^{C}$) to the models, which consists of a number of target dermoscopic lesion categories, indicated by the weight matrix *C*. This method of transferring pretraining provides a foundation network that can be generalized to heterogeneous dermatological databases as the pretrained weights can provide a good initialization that reduces the time taken for convergence and increases the robustness of the model when working with low-label datasets, while also allowing for the retention of the learn cross-lesion feature representations obtained in the self-supervised training environment.

### 3.3. Hierarchical Vision Transformer Backbone

The primary feature extraction component of the foundation model is the hierarchical vision transformer backbone which uses the ordered token sequence $X_{l}\in R^{N_{l}\times D_{l}}$ previously generated by the preceding stage(s) of processing to generate embeddings used within the framework’s multiple transformer blocks. For the purpose of this discussion, l∈{1,2,…,L} is the index of a given transformer block; $N_{l}$ is the count of the number of currently active tokens at block index $D_{l}$, which indicates the corresponding embedding dimensionality. Token embeddings enter a transformer block as three parallel linear projected representations: a query representation, a key representation, and a value representation, all of which are necessary for the computation of attention. The query, key and value projections are each parameterized by trainable matrices (i.e., $W_{l}^{Q}\in R^{D_{l}\times D_{l}}$, $W_{l}^{K}\in R^{D_{l}\times D_{l}}$, and $W_{l}^{V}\in R^{D_{l}\times D_{l}}$) and all maintain dimensional representation consistency throughout computation in the attention process.

(8) $$Q_{l}=X_{l}W_{l}^{Q}$$

(9) $$K_{l}=X_{l}W_{l}^{K}$$

(10) $$V_{l}=X_{l}W_{l}^{V}$$

To improve representational diversity and capture multiple relational patterns among dermoscopic regions, the attention operation is decomposed into *H* parallel attention heads, where *H* is a fixed hyperparameter that controls the number of subspace projections. Each head processes projected tokens of dimensionality $d_{h}=D_{l}/H$ and independently computes token similarity scores, forming soft attention weight distributions. For the *h*-th attention head, where h∈{1,2,…,H}, the corresponding head output is computed using scaled dot-product attention, whereby inter-token relevance is quantified through normalized inner products between corresponding query and key projections, followed by weighted summation of the value tokens.

(11) $$head_{h}=Softmax\;\left(\frac{Q_{l}^{\left(h\right)}{\left(K_{l}^{\left(h\right)}\right)}^{T}}{\sqrt{d_{h}}}\right)V_{l}^{\left(h\right)}$$

The outputs of all *H* attention heads are concatenated along the embedding dimension to form a joint multi-head attention representation. This concatenated representation is subsequently mapped through an output projection matrix $W_{l}^{O}\in R^{D_{l}\times D_{l}}$ to restore the original embedding dimensionality and integrate cross-head interactions into a unified attention response tensor denoted by $MSA(X_{l})$.

(12) $$MSA\left(X_{l}\right)=Concat\left(head_{1},\ldots ,head_{H}\right)W_{l}^{O}$$

To improve the stability of training deep transformer networks and ensure that gradients flow through deep stacks, we apply residual connections to the output of the Multi-Head Attention operation. At this point, we obtain the first stage of refinement, in which the original representation of each token has been incorporated into its context through an initial normalization applied independently to each token embedding. We denote the application of this normalization operation across the token dimensions as LN(·), which is applied in order to ensure that each token embedding has a mean of zero and a variance of one.

(13) $${\tilde{X}}_{l}=X_{l}+MSA\left(LN\left(X_{l}\right)\right)$$

After being passed through an attention-augmented token representation denoted by ${\tilde{X}}_{l}$ from the previous step, a Position-Wise Feed-Forward Network, denoted by MLP(·), was constructed using two fully connected layers separated by nonlinearly activated functions for the purpose of performing a transformation of the token’s features at a non-linear level. By this means, Lesion Descriptors are further refined by modelling higher-order feature interactions which cannot be captured solely through linear attention mechanisms.

(14) $$X_{l+1}={\tilde{X}}_{l}+MLP\left(LN\left({\tilde{X}}_{l}\right)\right)$$

**Figure 2:**
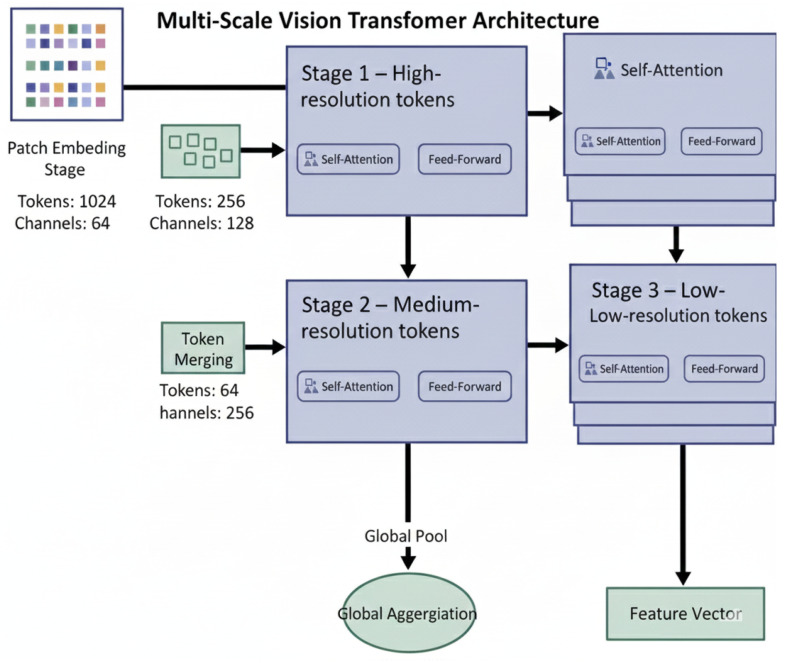
Architecture of the hierarchical Vision Transformer backbone.

To enable the use of high-resolution dermoscopic images while minimizing overall resource consumption, the hierarchical token reduction mechanism within the backbone architecture accumulates patch data across multiple levels of the network hierarchy. Merging tokens results in reducing the number of tokens ($N_{l}$), but increases the size of each token’s feature vector ($D_{l+1}$), allowing the model to create multiscale representations of local pigment micro-structures as well as global morphology patterns present in lesions. At the conclusion of the final transformer layer (*L*), a global summarization operation is performed on the final output sequence of tokens ($X_{L}$) resulting in the creation of an efficient lesion-level representation vector $f\in R^{D_{final}}$, where $D_{final}$ represents the highest level of the output feature vector size from the backbone. The global feature vector (*f*) is then used as the main diagnostic descriptor that is passed to the downstream classification head discussed in the overview section. The global summarization operation thus serves as a critical bridge between hierarchical attention and the supervised prediction of dermoscopic lesions within the foundation model pipeline. The internal architecture of the hierarchical Vision Transformer backbone, serving as the foundation model, is depicted in Figure 2.

### 3.4. Self-Supervised Pretraining Strategy

To enhance the generalization capacity and cross-domain robustness of the hierarchical transformer backbone introduced in the previous subsection, the proposed framework employs a self-supervised pretraining strategy that learns transferable visual representations directly from large-scale unlabeled dermoscopic datasets. Let the unlabeled dataset be defined as $D_{u}=X_{i}{i=1}^{M}$, where each element $X_{i}\in R^{H\times W\times 3}$ represents a normalized dermoscopic image sampled independently from diverse acquisition devices, patient populations, and lesion conditions, and *M* denotes the total number of unlabeled samples available for pretraining. For each image $X_{i}$, stochastic data augmentations are applied to generate multiple complementary views, including random spatial cropping, horizontal and vertical flipping, color jittering, brightness perturbation, and contrast shifting, thereby constructing view pairs that preserve semantic content while introducing controlled appearance variability.

These augmented samples are independently processed through the hierarchical backbone described in Section 3.3 to generate token embeddings $X_{L}^{\left(1\right)}$ and $X_{L}^{\left(2\right)}$ at the final transformer stage *L*, which are subsequently reduced via global aggregation to produce compact feature representations $h_{i}^{\left(1\right)}\in R^{Dfinal}$ and $h_{i}^{\left(2\right)}\in R^{D_{final}}$. The goal of self-supervised pretraining is to align feature representations derived from multiple augmented views of the same image while simultaneously discouraging feature similarity across distinct images, thereby enforcing instance-level discrimination and preventing degenerate representation collapse.

In reconstruction-based pretraining settings, the model is trained to recover masked input information from incomplete token sequences, thereby enforcing spatial reasoning and global contextual inference. A random subset of patch tokens indexed by the masking operator $\Omega_{i}\subset 1,2,\ldots ,N$ is removed from the input token matrix $X_{0}^{\left(i\right)}$, and the backbone attempts to reconstruct the original token contents from the remaining visible tokens. Let ${\hat{X}}_{i}^{mask}\in R^{|\Omega_{i}|\times 3P^{2}}$ denote the reconstructed flattened patch vectors corresponding to the masked token indices, and let $X_{i}^{mask}$ represent the ground-truth flattened patch values extracted from the original image. The reconstruction objective penalizes the mean-squared deviation between predicted and true patch content across all masked patches and all images in $D_{u}$.

(15) $$L_{rec}=\frac{1}{M}\sum_{i=1}^{M}{\left|{\hat{X}}_{i}^{mask}-X_{i}^{mask}\right|}_{2}^{2}$$

In parallel with reconstruction learning, contrastive instance discrimination is employed to encourage representational consistency across different augmented views of the same image while separating representations of different images. Let $h_{i}^{\left(1\right)}$ and $h_{i}^{\left(2\right)}$ represent the normalized embeddings produced by two distinct views of image $X_{i}$, and let $h_{j}$ represent the embedding of any image $X_{j}\neq X_{i}$ used as a negative sample within a mini-batch. Cosine similarity is utilized to compute dot-product affinities between embedding vectors. A temperature parameter τ>0 is introduced to modulate the concentration of the resulting similarity distribution. The contrastive loss is defined as a normalized cross-entropy objective that maximizes positive pair similarity while suppressing correlations with negative pairs.

(16) $$L_{con}=-\frac{1}{M}\sum_{i=1}^{M}\log\left(\frac{\exp\left(\frac{h_{i}^{\left(1\right)}\cdot h_{i}^{\left(2\right)}}{\tau }\right)}{\sum_{j=1}^{M}\exp\left(\frac{h_{i}^{\left(1\right)}\cdot h_{j}}{\tau }\right)}\right)$$

The total self-supervised pretraining objective integrates reconstruction and contrastive learning, thereby enforcing complementary structural constraints on feature learning. Weighting coefficients $\lambda_{rec}\geq 0$ and $\lambda_{con}\geq 0$ are introduced to balance the relative influence of the two objectives during optimization. The combined loss function driving self-supervised backbone training across the unlabeled dataset $D_{u}$ is therefore formulated as

(17) $$L_{ssl}=\lambda_{rec}L_{rec}+\lambda_{con}L_{con}$$

**Figure 3:**
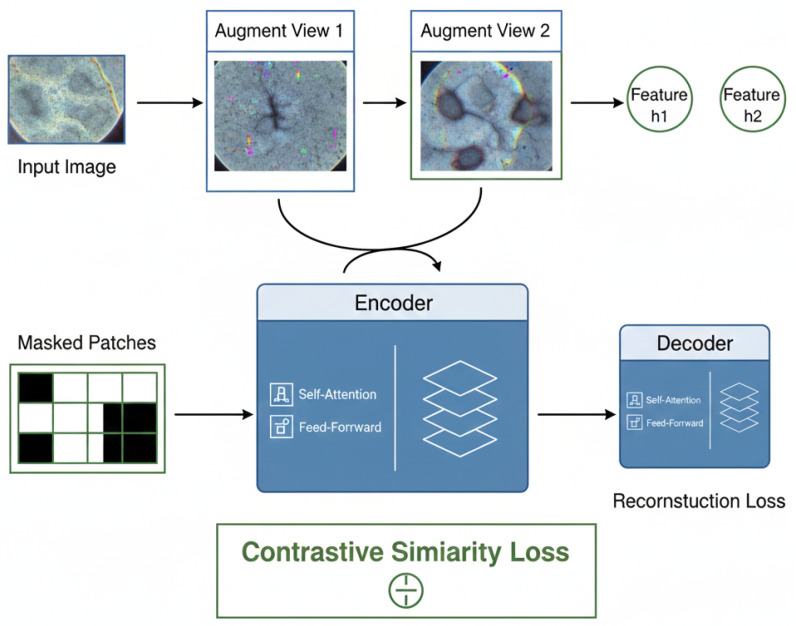
Self-supervised pretraining strategy for the proposed foundation model.

Minimizing the objective function $L_{ssl}$ with respect to the backbone parameters ensures learning hierarchical token representations that embed both spatial-structural consistency via reconstruction learning and instance discriminability via contrastive alignment. Upon convergence of the self-supervised training stage, the optimized backbone weights are preserved and transferred directly into the supervised fine-tuning phase described in the subsequent subsection, enabling effective knowledge reuse without re-initialization and providing a powerful initialization that substantially stabilizes downstream training under limited labeled data availability and pronounced cross-dataset distribution shifts. The joint masked reconstruction and contrastive learning scheme used during self-supervised pretraining is illustrated in Figure 3.

During self-supervised pretraining, the goal is to learn lesion-scale visual representations that are both invariant to data augmentations and maintain spatial and morphological coherence across augmentations. The model achieves this by optimizing the combined objective defined in Equation (12); this objective combines the goals defined in Equations (10) and (11), which are described below. The objective of the reconstruction component is to predict masked patch tokens from the surrounding context, encouraging the learning of how lesions can form spatial structure and continuity within the overall morphology. This helps learn more anatomically meaningful representations of the features captured in lesion images. The active branch uses different augmentations of the same dermoscopic image to define positive pairs and different dermoscopic images to define negative pairs.

By maximizing agreement between positive pairs and minimizing agreement between negative samples, the model learns to discriminate between image-level representations that can be affected by appearance changes, while simultaneously distinguishing between images of different lesions. By optimizing both the reconstruction and contrastive objectives simultaneously, the backbone of the model learns transferable dermoscopic representations that encompass both local coherence structurally and the global ability to differentiate between lesions at the instance level. Thus, the self-supervision phase creates a representation space where images of the same lesion cluster under augmentation, while also allowing the structural and semantic separation of distinctly different lesions, providing a strong basis for future supervised fine-tuning under domain shift and limited-labeled conditions.

#### 3.4.1. Self-Supervised Pretraining Data and Ethical Considerations

The proposed foundation model’s self-supervised pretraining was performed using a large number of unlabeled dermatological images gathered from existing public dermatology repositories, collected by organizations and institutions that gather and make data publicly available. Most of these images were from the ISIC 2018 Dataset, while a small but significant number came from the HAM10000 and PH2 Datasets. These datasets contain a variety of skin ALS types and were acquired using different protocols. Each of the three datasets contains different types of lesions, photographs taken with different equipment, and under varying conditions. The ISIC 2018 Dataset contains more images (over 10,000) than the other two datasets, and it also includes multi-class lesion-annotated dermoscopy photographs that are more than 10 years old.

The HAM10000 Dataset contains more than 10,000 dermoscopy images from numerous clinical centers worldwide and exhibits variability in illumination, resolution, and appearance. The PH2 Dataset is significantly smaller than both the ISIC 2018 and HAM10000 Datasets, but it contains high-resolution dermoscopic photos shot with similar equipment under the same conditions. Together, these images help expand the overall representational space by providing additional visual characteristics to compound dermoscopy images. Each of the images that underwent self-supervised pretraining in this research project passes through the same preprocessing procedure, which includes resizing to a single spatial resolution for all images, normalizing each channel and applying stochastic augmentations such as random selection of cropped areas for the image, reverse mirroring (flipping), light variance (brightness/contrast), flourished (modified) and mild-affine (mildly distorted) transformations.

Ethically, the data that was utilized for this research project consisted only of the three datasets referenced and used in accordance with the data provider’s permissive license, and thus, no identifying information regarding any of the patients in this study’s dataset was available to either the authors of this research project or any others who may read this paper. Since the data collected and released in the datasets used in this research project have already been rendered unidentifiable, there was no requirement to obtain Institutional Review Board authorization or the consent of the patients involved in using these datasets. Data from this research project will be used only under the conditions established by the data provider.

#### 3.4.2. Self-Supervised Masking Strategy and Pretraining Objectives

In the process of self-supervised pretraining, the two techniques, masked image modeling and contrastive instance discrimination, are combined to build a strong, generalizable, and transferable representation of an input image. For every image used in this pretraining process, a 40% mask of the patches was generated randomly from pixel indices in the dermoscopic image, without accounting for the patch structure. Because both types of masking are random patterns, they will require the model to understand both textural information from local patches and context related to a larger view of the image, using all visible patches. Each training image and each version or augmented view of that image will be masked with a different pattern, so any fixed token delimitation is avoided; each will be masked and rebuilt randomly, at the cost of visual coherence for each image. supervised pretraining objective is incorporated through a combined loss function.

The masked-image reconstruction loss minimizes the squared differences between the patch embeddings produced by the masked and original images. This constraint forces the model to think spatially and builds upon its ability to learn a structural representation of the images used for training. Also concurrently with the masked reconstruction loss, a contrastive loss function based upon the normalized temperature-scaled cross entropy and from the derived representations of the same and different images using different augmented versions of those images will cause the model’s embedding equations to align in an invariant view while also encouraging a collapse of embeddings from distinct images. The self-supervised loss for each pretraining trial is computed as the sum of the two separate losses, each multiplied by a constant weight, allowing the foundation model to learn, simultaneously, local features and globally relevant descriptors prior to supervised adaptation.

Self-supervised training uses only dataset images from ISIC 2018, HAM10000, and PH2 that were generated from each dataset’s training splits, eliminating leakage from validation/test images and providing a fair assessment of the learning process. Images that came from either the validation/test splits of the dataset were not used at this point, whether labeled or not, and in addition, all splits used in the self-supervised learning process keep the level of separation of patients intact; therefore, each patient’s images will not appear throughout the final experiment, as all images will have remained distinct throughout the entire training. Since self-supervised training does not use diagnostic labeling, this approach allows the representations produced by the self-supervised process to be completely independent of test-set distributions and, therefore, to preserve the validity of cross-dataset generalization results.onTask-Specific Fine-Tuning for Dermoscopic Lesion Imaging.

After completion of the self-supervised pretraining stage described in Section 3.4, the pretrained hierarchical transformer backbone is adapted to supervised diagnostic classification tasks using labeled dermoscopic imaging datasets. Let the labeled dataset be denoted by $D_{l}=\left(X_{i},y_{i}\right){i=1}^{N_{l}}$, where each element consists of an image sample $X_{i}\in R^{H\times W\times 3}$ and its associated diagnostic class label $y_{i}\in 1,2,\ldots ,C$, with *C* representing the total number of disease categories included in the classification task and $N_{l}$ denoting the total number of training instances. Each image $X_{i}$ is processed by the pretrained backbone network to yield a compact global lesion feature representation $f_{i}\in R^{D_{final}}$, where $D_{final}$ denotes the embedding dimensionality produced by the final aggregation stage of the hierarchical transformer described previously.

This representation encapsulates multiscale contextual lesion information, including pigmentation variability, boundary irregularity, and shape deformities learned during unsupervised representation learning. To translate latent feature embeddings into categorical disease predictions, a classification head parameterized by a learnable weight matrix $W_{c}\in R^{C\times D_{final}}$ and bias vector $b_{c}\in R^{C}$ is appended to the backbone. The head computes a linear transformation of the features to generate class-specific logits $o_{i}\in R^{C}$, where each element $o_{i}^{c}$ represents the normalized activation score for the *c*-th disease category associated with image $X_{i}$.

(18) $$o_{i}=W_{c}f_{i}+b_{c}$$

The computed logit vector $o_{i}$ is subsequently converted to normalized class membership probabilities via the softmax function, yielding a categorical distribution over the *C* disease classes for each sample. The resulting probability value $p(y=c|X_{i})$ reflects the model-estimated likelihood that image $X_{i}$ belongs to diagnostic category *c*, subject to normalization across all class labels to ensure $\sum_{c=1}^{C}p\left(y=c|X_{i}\right)=1$ for every sample.

(19) $$p\left(y=c|X_{i}\right)=\frac{\exp(o_{i}^{c})}{\sum_{k=1}^{C}\exp\left(o_{i}^{\left(k\right)}\right)}$$

During fine-tuning, both the transformer backbone parameters inherited from the pretraining stage and the classification head parameters $\left(W_{c},b_{c}\right)$ are optimized jointly using labeled data from $D_{l}$ according to supervised learning objectives detailed in the subsequent optimization subsection. Fine-tuning updates allow the pretrained representations to realign with disease-specific discriminative boundaries and subtle dermatological patterns present in annotated datasets while preserving the general-purpose features captured during the self-supervised stage. Pixel-level and color-based data augmentation strategies, including random cropping, geometric distortion, intensity variation, and color perturbation, are employed during supervised training to improve domain robustness and encourage model invariance to acquisition conditions commonly observed in real clinical imaging workflows. Because the backbone is pretrained on heterogeneous unlabeled sources, supervised fine-tuning can be performed independently across datasets with distinct class definitions and sample distributions, enabling the proposed framework to operate as a modular, plug-and-play feature extractor adaptable to future multi-class diagnostic tasks without architectural redesign or re-initialization.onOptimization Objective and Training Protocol.

The supervised adaptation of the pretrained foundation model described in Section 3.4.2 is guided by an optimization objective that aligns predicted disease probabilities with ground-truth diagnostic labels while preserving the representational stability inherited from the self-supervised stage. Let a mini-batch of labeled samples be denoted by $B\subset D_{l}$ with cardinality |B|, where each batch element consists of an image-label pair $\left(X_{i},y_{i}\right)$, $X_{i}\in R^{H\times W\times 3}$ representing a preprocessed dermoscopic image and $y_{i}\in 1,2,\ldots ,C$ indicating its categorical disease class index. For each sample $X_{i}$, the classification head outputs a probabilistic prediction vector computed through the softmax mapping described previously, yielding $p(y=c|X_{i})$ for all class indices *c*. The categorical cross-entropy loss function quantifies the discrepancy between predicted probabilities and ground-truth labels by penalizing log-likelihood deviation across the mini-batch. This objective maximizes the likelihood of correct class assignments during fine-tuning.

(20) $$L_{ce}=-\frac{1}{\left|B\right|}\sum_{(X_{i},y_{i})\in B}\log p\left(y_{i}|X_{i}\right)$$

To address the class imbalance common in dermatological datasets, where certain lesion categories exhibit significantly lower prevalence than others, a weighted variant of the cross-entropy objective is used. This strategy introduces class-dependent scaling coefficients $\alpha_{c}>0$ for each class label c∈1,2,…,C, which amplify gradient contributions from minority classes and attenuate those from more frequent categories. Denoting $\alpha_{y_{i}}$ as the weight assigned to the ground-truth class corresponding to sample $X_{i}$, the weighted categorical loss applied at the batch level is formulated as follows.

(21) $$L_{wce}=-\frac{1}{\left|B\right|}\sum_{(X_{i},y_{i})\in B}\alpha_{y_{i}}\log p\left(y_{i}|X_{i}\right)$$

The final supervised training objective integrates the weighted classification loss and regularization terms designed to stabilize learning and prevent overfitting of the backbone transformer weights and classification head parameters. Regularization is represented by the term $L_{reg}$ and encompasses decoupled weight decay applied to all network parameters, label smoothing mechanisms that reduce overfitting in classification confidence, and structured stochastic depth within transformer blocks to enhance model generalization by implicitly ensembling sub-networks during learning. The scalar coefficient $\lambda_{reg}\geq 0$ controls the relative contribution of regularization to the overall objective and is tuned empirically based on validation performance.

(22) $$L_{total}=L_{wce}+\lambda_{reg}L_{reg}$$

To mitigate the effects of class imbalance common in dermoscopic datasets, a class-weighted categorical cross-entropy loss is employed during supervised fine-tuning. Class weights are computed from the inverse class frequencies in the training data, ensuring greater sensitivity to underrepresented lesion categories while preventing dominance by the majority classes. Optimization of both self-supervised pretraining and supervised fine-tuning objectives is carried out using the AdamW optimizer, which incorporates adaptive moment estimation with decoupled weight decay to ensure stable convergence across large-scale transformer architectures. A notation for the learning rate at iteration *t* during training will be denoted as: $\eta_{t}$. In contrast, during self-supervised pre-training, $\eta_{t}$ is held to a larger initialization to support rapid creation of large-scale representations/feature learning across large amounts of ‘unlabeled’ data; during this time, we also process mini-batches in much larger sizes than we would for supervised training, to stabilize the contrastive discriminative and also the masked token reconstruction objectives. To address class imbalance, weighted categorical cross-entropy is employed during supervised fine-tuning. Class weights are computed solely from the training set using inverse class-frequency statistics. Specifically, for each class *c*, the corresponding weight $w_{c}$ is defined as

(23) $$w_{c}=\frac{N}{C\cdot n_{c}},$$

where *N* denotes the total number of training samples, *C* is the number of classes, and $n_{c}$ represents the number of training samples belonging to class *c*. This weighting strategy increases the contribution of underrepresented lesion categories during optimization while maintaining stable gradient magnitudes across classes.

The optimization objective during supervised fine-tuning is defined by Equations (15)–(17). The first part of this objective is the categorical cross-entropy loss defined in Equation (15), while Equation (16) will be used for a class-weighted version of the loss to better represent minority classes in dermoscopy datasets, which are imbalanced. Class weighting is determined by taking the inverse of the class’s proportion in the training set. This means minority class labels will be given higher weights in the optimization process than majority class labels. The final part of the objective defined in Equation (17) takes the class-weighted loss computed in Equation (16) and combines that with the regularization terms applied to the loss, which are a result of using label smoothing and stochastic depth. Label smoothing is applied to target distributions to soften them and reduce overconfident predictions, while stochastic depth is a design feature of the architecture that helps the optimization process by randomly removing transformer layers during training, which implicitly regularizes the optimization.

By jointly optimizing these two components without modifying the base loss, we will improve generalization and the stability of the training process.o through to the supervised fine-tuning stage, we will therefore use significantly less values for learning rates and also substantially lower batch sizes, in order to preserve the feature space of the pre-trained foundation model and at the same time to fine-tune the class ‘boundaries’. Learning rate behavior is driven by either Cosine Annealing or Piecewise Step Decay, providing a very consistent overall and a smooth, but monotonic, decline throughout the entire training run. The same set of data augmentations is applied consistently throughout both phases, in particular geometric cropping, flipping about the horizontal and vertical axes, pigment intensity modulation, jittering across multiple channels, and small affine transformations to provide invariance to variability in the source and the ‘imaging device’. Through this overall training method, the developed models are a stable transformer-based foundation that can learn the global structure of dermatological conditions and fine-tune for disease-specific classification tasks during the supervised phase of training.

## 4. Results

In this section, we describe the experimental studies conducted to evaluate our proposed transformer-based foundation model, in accordance with the training and optimization protocol we established. Three publicly available dermoscopic databases (ISIC 2018, HAM10000, and PH2) were used to evaluate the performance of our proposed model, as they differ in class imbalance ratios, lesion diversity, and imaging acquisition characteristics. The same preprocessing pipelines and data augmentation policies were used to ensure a consistent evaluation and comparison across all three datasets. Multiple training and evaluation runs were performed on various random splits to help eliminate stochastic variance and enhance reproducibility for the trend data. The results of these studies have been evaluated from three main areas of view: examples of the classification accuracy that occurred within the datasets, evidence that our proposed model is able to generalize across datasets, as it demonstrated strong performance when tested outside the original database, and the performance of the model with limited data when it is trained with low annotated clinical situations. All of the analyses conducted will be referenced in the following subsections, along with our figures, which will be submitted for publication with this manuscript.

### 4.1. Datasets and Experimental Setup

The three dermoscopic image datasets used for the experimental evaluations in this research are openly available and are widely considered the gold standard for developing algorithms that use artificial intelligence in dermatology. These datasets are ISIC 2018, HAM10000, and PH2. ISIC 2018 includes a large collection of dermoscopy images for clinical diagnosis, labeled with multiple diagnostic categories, covering both benign and malignant lesions, and obtained using various clinical imaging devices under various conditions. The images produced by ISIC 2018 present an extreme imbalance in so-called inter-class variability, which is representative of the actual class distribution of the dermatological community and provides a highly rigorous method of evaluating the generalizability of dermoscopic algorithms trained using a framework that was not built to account for sparsity within minority classes or the complexities involved with diagnosing these conditions.

The HAM10000 dataset was created from images collected from dermatology departments worldwide, using a wide variety of devices, image resolutions, and illumination conditions, to facilitate a thorough evaluation of what it means to generalize from a multi-source to a single-source domain. The PH2 dataset contains standardized high-resolution dermoscopy images captured under extremely similar conditions and annotated in a controlled clinical environment, making them ideal for investigating the impact on performance and generalizability when an algorithm is trained across multiple data sources. Before training an algorithm, all three datasets (ISIC 2018, HAM10000, and PH2) are resized to a common spatial resolution and normalized channel-by-channel to ensure compatibility with the unified training pipeline. When a dataset contains information about individual patients, it is split at the patient level, thereby eliminating potential leakage of patient identifiers across training, validation, and test sets and ensuring that the algorithm’s performance is not biased.

For all datasets, data partitioning is done at the patient level to prevent images from the same patient from appearing in multiple splits. Stratified fixed ratios ensure class distributions are preserved across training, validation, and test sets. The validation set serves exclusively for model selection and early stopping, whereas the test set remains unseen until final evaluation. All reported results use a single fixed split per dataset, ensuring consistency and fair comparisons across methods. Table 2 provides a summary of this protocol.

The protocol followed during experimentation ensures that training was consistently conducted under unified, reproducible conditions across all datasets and baseline models. All datasets will have equal distribution across the train, validate, and test sets, with their partitions stratified using a fixed-ratio split to ensure equal class counts in each partition. Patients will be kept isolated within each partition, and thus, no patient data from the validation/test partitions were included in the training pool of images to avoid possible evaluation leakage from the validation/test sets. After pretraining the transformer backbone (using self-supervised learning) on a pool of only the training (unlabeled) images, fine-tuning the transformer model (using supervised learning) will occur for each individual dataset, using only the labeled images from that dataset (the training partition). The performance of all models will be determined using ONLY batch-wise validation, without using any of the validation/test partitions to determine hyperparameters, stopping criteria, or the learning rate.

All models were trained using the AdamW Optimizer, which uses weight decay on a decoupled basis. The learning rate for supervised pretraining is $3\times 10^{-4}$, and during fine-tuning, it is set to $1\times 10^{-4}$ to facilitate stabilization of feature adaptation. Batch sizes for pretraining are 256 and for fine-tuning are 64, which will ensure that convergence occurs quickly and meet the constraints of available memory. Training is performed for up to 300 epochs during pretraining and 100 epochs during fine-tuning, with early stopping applied using a patience window of 15 validation epochs. Weight decay is fixed at $1\times 10^{-2}$, dropout probability is set to 0.1, and stochastic depth with a maximum drop rate of 0.2 is applied to transformer layers to reduce overfitting. Data augmentation strategies, including random cropping, horizontal and vertical flipping, color jittering, brightness shifts, and mild affine distortions, are consistently applied only to training samples, whereas validation and testing data remain unperturbed to reflect real-world deployment settings. All experiments are executed on identical GPU infrastructure to ensure comparable run times and training stability across comparative evaluations. A summary of all hyperparameters and implementation settings used in the experimental protocol is provided in Table 3 for full reproducibility.

### 4.2. Training Configuration and Reproducibility Details

All experiments on the four aspects of self-supervised pretraining and supervised fine-tuning were conducted under a single, reproducible training setup. Specifically, during self-supervised pretraining, models were trained with the AdamW optimizer at a starting learning rate of $3\times 10^{-4}$, using cosine annealing as the learning rate scheduler, and a batch size of 256 for 300 epochs. Supervised fine-tuning was conducted with an averaged learning rate of $1\times 10^{-4}$ and a batch size of 64 for 100 epochs, while early stopping was employed based on validation performance with a patience window of 15 epochs. Weight decay was constant at $1\times 10^{-2}$ for all experiments. All models also employed 0.1 dropout and stochastic depth, with maximum dropout rates up to 0.2 within the transformer layers to help counteract overfitting. To address class imbalance during supervised training, a weighted cross-entropy loss was used, with class weights calculated as the inverse of each class’s frequency in the training data set. All experiments were run on identical GPU hardware to ensure fair comparisons and consistent runtime across all data sets and baseline models. The reported results are the means and standard deviations across five runs with different random seeds and reflect the robustness of each experiment to stochastic variation. During self-supervised pretraining and hyperparameter tuning, the validation and test sets were not included; thus, all hyperparameters were verified and consistent prior to evaluation, thereby preventing the risk of information leakage.

#### Comparison Scope and Model Selection Justification

The purpose of this work is to provide additional context on the use of foundation learning in dermatology by presenting examples and references to recently proposed models, such as DINO-based self-supervised learning frameworks, Med-ViT, and Med-SAM, as well as multimodal solutions like SkinGPT-4. Unfortunately, due to significant differences in how the models define the task(s) being evaluated, their overall design, and how they are evaluated under different protocols, it is not possible to perform any type of experimental comparison between them, as was done for the models developed in this study. Most dermatological foundation models developed using DINO-based self-supervised learning frameworks were evaluated using representation learning or linear probing methods, and as a result, they typically do not include publicly released, standardized pre-trained weights or end-to-end classification pipelines for benchmarking across multiple datasets. Med-SAM and related models were developed as segmentation-oriented foundation models and, as such, are optimized to generate prompt-based masks to segment images on a per-mask basis rather than to classify lesions with exact percentages of accuracy. Similarly, the multimodal solutions, such as SkinGPT-4, were developed to assist users with diagnostic recommendations in an interactive manner and to incorporate large amounts of publicly available clinical information. This is why SkinGPT-4 cannot produce reproducible image-only classifications. The primary purpose of this research project is to develop a new dermatology-specific, image-centric transformer foundation model optimized for robust multi-class lesion classification under in-dataset, cross-dataset, and limited-labeled conditions using only public data. As a result, all comparisons in this study are restricted to state-of-the-art transformer- and hybrid-based models, as no other models of this type are available and offer the greatest opportunity for fair and reproducible results.

### 4.3. In-Dataset Classification Performance

**Figure 4:**
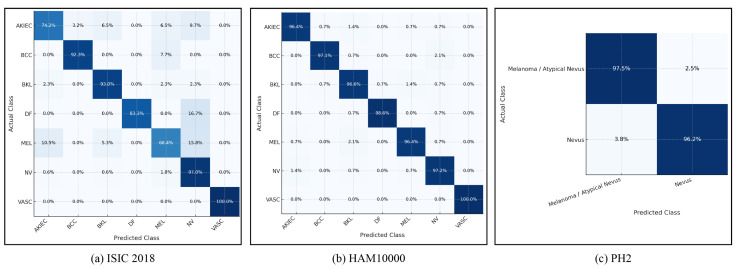
Normalized confusion matrices (%) of the proposed foundation model across the three evaluation datasets: (**a**) ISIC 2018 multi-class lesion classification; (**b**) HAM10000 multi-class lesion classification; (**c**) PH2 binary melanoma versus nevus classification.

The in-dataset classification performance evaluates the diagnostic capability of the proposed Transformer-Based Foundation Model when self-supervised pretraining and supervised fine-tuning are carried out entirely within the same dataset domain. Independent experiments are conducted for ISIC 2018, HAM10000, and PH2 using standardized training, validation, and test splits aligned with the protocols described in Section 4.1. Comparisons are performed against representative state-of-the-art baselines reported in recent literature. Across all datasets, the proposed foundation model consistently demonstrates higher classification accuracy, stronger macro-averaged F1-score stability, and greater sensitivity to minority lesion categories than competing methods. These improvements are particularly evident for lesion types characterized by complex chromatic variation and irregular boundary morphology, highlighting the benefits of hierarchical attention modeling and domain-specific foundation pretraining for capturing multiscale contextual relationships that remain difficult for conventional supervised architectures. The class-wise prediction behaviour of the proposed foundation model on ISIC 2018, HAM10000, and PH2 is visualized using normalized confusion matrices in Figure 4.

Table 4 presents comparative in-dataset results on ISIC 2018 against recent transformer-based and hybrid baselines. While all attention-driven architectures outperform classical convolutional designs, the proposed foundation model achieves the highest overall accuracy, F1-score, and AUC, indicating superior diagnostic discrimination and improved robustness against dataset imbalance and inter-class visual similarity.

The in-dataset evaluation on HAM10000 shown in Table 5 further demonstrates the consistent advantage of the foundation model against leading ensemble-based and multimodal approaches. Despite the already strong performance of multimodal fusion networks and deep ensemble CNNs, the proposed model delivers superior accuracy and macro F1-score stability, suggesting improved utilization of pretraining-induced generalizable representations across lesion subtypes and imaging sources.

Results on the PH2 dataset are summarized in Table 6, comparing the proposed model with attention-augmented CNNs and segmentation-related hybrid methods. Despite the relatively small dataset and reduced diversity, the foundation model achieves the highest classification accuracy and F1-score stability, underscoring its resistance to overfitting and strong cross-sample generalization enabled by large-scale pretraining and hierarchical attention fusion.

### 4.4. Cross-Dataset Generalization

**Figure 5:**
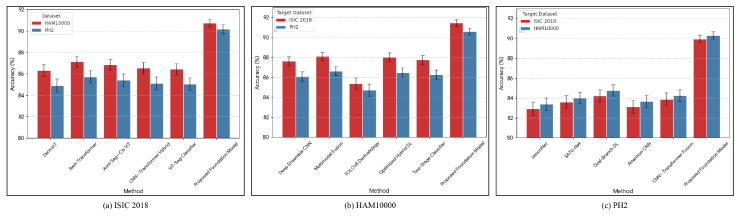
Comparison of mean classification accuracy (%) between the proposed foundation model and baseline methods: (**a**) Performance on the ISIC 2018 target dataset when pretrained on HAM10000 and PH2; (**b**) Performance on the HAM10000 target dataset when pretrained on ISIC 2018 and PH2; (**c**) Performance on the PH2 target dataset when pretrained on ISIC 2018 and HAM10000.

The cross-dataset generalization study evaluates the robustness of the pretrained Transformer-Based Foundation Model when transferred to imaging distributions that differ in acquisition devices, annotation policies, lesion taxonomy, and population heterogeneity. In this evaluation protocol, large-scale self-supervised pretraining is conducted on pooled unlabeled data across all datasets, followed by supervised fine-tuning on a single source dataset and testing on a distinct, unseen target dataset without any additional retraining or calibration. This experimental configuration effectively simulates real-world deployment scenarios in which diagnostic systems trained in one clinical domain must operate reliably across external clinical sites. A cross-dataset comparison against strong baseline methods in terms of mean accuracy and variance is reported in Figure 5.

Table 7 summarizes transfer performance when fine-tuning is conducted on ISIC 2018, and evaluation is performed on external test sets from HAM10000 and PH2. Traditional supervised transformer and hybrid CNN–Transformer baselines, such as DermViT [24], and Swin Transformer [25], experience substantial degradation due to acquisition and population shifts, while the proposed foundation model maintains substantially higher classification accuracy and AUC stability across both target datasets, confirming the benefit of large-scale unlabeled dermatology-specific pretraining.

When HAM10000 serves as the training source dataset, Table 8 reports target-domain testing on ISIC 2018 and PH2. Ensemble CNN architectures [5] and multimodal fusion systems [28] demonstrate modest improvements in stability relative to single-backbone networks, yet their reliance on labeled-domain statistics limits true cross-site generalization. In contrast, the proposed foundation model achieves the highest target-domain classification accuracy and the highest overall AUC consistency across both test datasets, underscoring the importance of foundation-scale pretraining for learning transferable lesion morphology representations independent of site-specific imaging biases.

When PH2 serves as the supervised fine-tuning source dataset, Table 9 evaluates generalization performance on ISIC 2018 and HAM10000. PH2-trained baseline models such as SATU-Net [33], dual-branch segmentation-classification systems [34], and attention-guided CNN frameworks [35] suffer marked drops in transfer accuracy due to small dataset size and limited lesion diversity. In contrast, the proposed foundation model remains substantially less sensitive to these limitations, maintaining stable classification accuracy and high AUC values across both large-scale target datasets, thereby highlighting the unique ability of foundation-scale representation learning to overcome limited source data regimes.

A strict separation of datasets when performing cross-dataset transfer experiments enables an unbiased evaluation of models trained on a source dataset. The model is trained on a specific portion of the source dataset for validation and early stopping. All validation and early stopping are performed on the target datasets using only the source dataset’s training split. The target dataset will not contain any labeled information before training, validation, or hyper-parameter selection; rather, it will serve as an entirely unseen domain used solely for final test results. No examples from the target dataset have been used in training or validation. There has been no adaptation or fine-tuning based on the target domain labels. By using this protocol, cross-dataset outcomes can be interpreted as the model’s actual performance when generalized to a different domain, without any implicit adaptation or information leakage.

### 4.5. Cross-Dataset Error Analysis and Class-Wise Performance Trends

The analysis of failure modes and class-level performance trends using normalized confusion matrices in Figure 4. We use normalized confusion matrices to examine class-level failure modes and trends in our proposed foundation model, enabling us to better assess its generalizability and robustness. All three data sets contain many instances of lesion categories that exhibit substantial visual heterogeneity and distinct morphological characteristics, such as melanoma and seborrheic keratosis. For these lesion categories, the proposed foundation model pretraining approach appears to improve performance by increasing diagonal dominance in the respective confusion matrices and, consequently, improving sensitivity and reducing inter-class confusion, particularly when evaluated on cross-data sets.

In contrast, benign lesions that visually resemble one another and share similar pigmentation and texture characteristics exhibit substantial inter-class confusion across all datasets. These misclassifications are likely to arise from intrinsic diagnostic ambiguity rather than from model instability. In addition to class-level misclassifications, we have also observed that many failure cases occur near class boundaries between lesions that are visually similar but exhibit subtle differences in appearance, such as atypical nevi versus benign nevi. This increase in misclassifications near class boundaries is likely due to greater visual appearance variation resulting from shifting to a different domain. Such examples align with known challenges in interpreting dermoscopic images and suggest that residual confusion is likely due to intrinsic visual appearance overlap rather than dataset overfitting.

Although explicit demographic information is not available for all datasets, sample biases arising from acquisition-related factors were indirectly explored in cross-dataset transfer experiments. Performance decreases on external datasets for baseline models indicate increased sensitivity to differences in clinical imaging devices, clinical illumination conditions, and clinical protocols. However, the proposed foundation model demonstrates substantially higher stability in performance across multiple datasets than baseline models, indicating a reduced dependence on dataset-specific acquisition factors.

### 4.6. Limited-Data Fine-Tuning Analysis

To investigate the effectiveness of self-supervised dermatology-specific pretraining under clinically realistic low-annotation scenarios, we conducted a limited-data fine-tuning analysis. In this analysis, supervised training was restricted to progressively smaller fractions of labeled samples while maintaining fixed validation and testing partitions. Experiments were performed across all three evaluation datasets (ISIC 2018, HAM10000, and PH2). Fine-tuning subsets comprised 10%, 25%, and 50% of the available training data.

Table 10 summarizes the low-label fine-tuning results on the ISIC 2018 dataset. Under the most constrained training regime using only 10% labeled samples, baseline transformer-based methods [24,25] suffer notable performance degradation, whereas the foundation model maintains comparatively high classification accuracy and stability across training runs. The observed performance gap gradually narrows as labeled data becomes available, yet the proposed method continues to outperform all baselines even when half of the training data is used.

The limited-data fine-tuning outcomes on the HAM10000 dataset are reported in Table 11. Ensemble CNN classifiers [5] and multimodal fusion networks [28] demonstrate improved stability relative to single-backbone learners in low-label regimes; however, they are consistently surpassed by the foundation model, especially when only 10% or 25% of the data are labeled. These results further confirm the critical role of representation pretraining for mitigating overfitting risks under severe annotation constraints.

Limited-data experiments conducted on the PH2 dataset are summarized in Table 12. In small-sample settings, model overfitting variability across training trials is typically greater for conventional attention-driven CNN models such as SATU-Net [33], and residual frameworks, including LesionNet [32]. In contrast, the proposed foundation model demonstrates substantially improved training stability and consistently elevated diagnostic accuracy across all labeling fractions, highlighting the ability of large-scale dermatology-specific self-supervised initialization to suppress variance propagation and enhance early-stage fine-tuning convergence under restricted annotation budgets.

### 4.7. Ablation Study

An ablation analysis is performed to systematically quantify the contributions of core architectural and training components of the proposed Transformer-Based Foundation Model. Evaluated configurations include removing self-supervised dermatology-specific pretraining while retaining only supervised fine-tuning, replacing the hierarchical multi-stage transformer backbone with a single-scale transformer architecture of equivalent depth, eliminating the token-merging modules responsible for progressive spatial down-sampling, and omitting advanced data augmentation strategies during supervised optimization. To achieve meaningful results and quantify performance across environments, an assessment is conducted using several methods to isolate outcomes from individual observations from each group. In every instance below, the most likely explanation for the decline in performance of each of these previously mentioned configuration changes occurred when self-supervised pretraining was excluded, further validating the critical role self-supervised pretraining plays in developing effective models to extract transferable representations of lesions. Performance degradation is pronounced when hierarchical token aggregation is no longer used. Performance degradation due to data augmentation is observed through reductions in model value, variability, and overall effectiveness.

The results presented in Table 13 relate directly to each independent configuration change for the ISIC 2018 dataset. The deletion of self-supervised pretraining results in the largest decrease in both classification accuracy and F1 score consistency, whereas replacing the hierarchical backbone with a single-scale transformer prevents efficient global–local feature fusion, thereby severely reducing the model’s efficacy. By removing token merging and using hierarchical aggregation to obtain token-level features, there is an additional reduction in multiscale contextual aggregation, which negatively influences sensitivity to ambiguous lesion boundaries. Furthermore, removing advanced data augmentation results in ongoing moderate decreases in performance during training trials, due to increased variance across multiple runs of the same trial(s). In conclusion, the overall results indicate that the full model configuration yields the best diagnostic performance across all evaluated metrics.

Table 14 provides a summary of the ablation study performed on HAM10000. In alignment with results from the ISIC dataset, it was observed that removing self-supervised pre-training had the greatest effect on drop-off in accuracy and on F1-score stability, attributed to a loss of robustness in the initialisation of dermatological representations. Both eliminating hierarchical backbone structures in favour of a single-scale transformer architecture and eliminating token-merging operations resulted in significant performance decreases, indicating the value of employing multiscale feature extraction when processing the various lesion subtypes found in expansive heterogeneous databases. Removing data augmentation led to modest but statistically significant performance decreases, reinforcing the importance of this mechanism for maintaining model robustness when data is acquired variably.

The ablation studies conducted with the PH2 dataset illustrated in Table 15 furthermore support the conclusion that the foundations learned via pretraining provide an essential basis for modelling under conditions of a limited training sample size. Self-supervised pretraining was responsible for the greatest loss of accuracy; the removal of hierarchical modelling mechanisms adversely affected the stability of specific feature extraction at the lesion boundary, while data augmentation reductions increased variance in performance, attributed to greater vulnerability to overfitting due to limited training samples. The complete foundation model demonstrated continued consistency and superior performance across multiple repeated experimental trials in this small but difficult-to-acquire clinical dataset.

### 4.8. Computational Efficiency and Convergence Behavior

**Figure 6:**
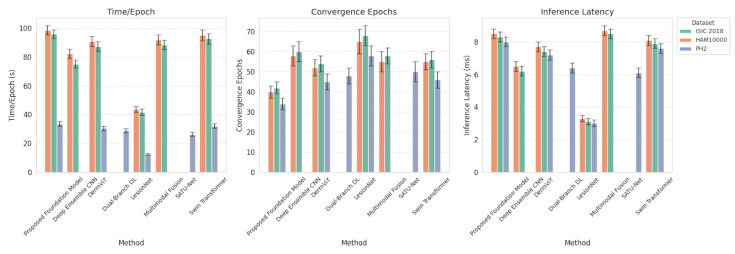
Computational efficiency analysis of the proposed foundation model compared to competing methods across ISIC 2018, HAM10000, and PH2: Left: average time per training epoch; Middle: number of epochs required to reach convergence; Right: inference latency per image (ms). Bars correspond to different datasets, and error bars denote standard deviation across repeated runs.

To determine the practicality of using the proposed Transformer-Based Foundation Model across different types of clinics, we assess both its computational efficiency and its convergence when combined with existing methodologies. Although lightweight CNN-based approaches, such as LesionNet, provide shorter training and inference times (i.e., fewer epochs), these models require significantly longer convergence times and many more iterations to attain optimal performance, especially when trained with limited data. The transformer’s computational load is generally much higher but exhibits better convergence characteristics than a traditional CNN model. In fact, the proposed Transformer Foundation Model had a slightly larger parameter footprint (approximately 96 million parameters) than most CNN-like architectures (ranging from roughly 18 to 65 million parameters) and traditional transformer baselines (approximately 85 to 90 million parameters). However, the proposed model consistently demonstrated the fastest convergence times across all datasets, with competitive inference latency, thereby providing an effective trade-off between training efficiency and implementation feasibility. Table 16 contains a comprehensive summary of the computational efficiency of the different models across the various datasets. The computational efficiency of the proposed foundation model in terms of training time, convergence speed, and inference latency is summarized in Figure 6.

### 4.9. Positioning Within Dermatology-Oriented Foundation Models

Recently, dermatology-oriented foundations have become more widely available in a wide variety of designs, including large-scale self-supervised representation learning, segmentation-oriented promotable systems, and a multimodal diagnostic assistance system. The proposed work proposes a unique design documentation position based on several aspects, namely pre-training methods, architecture re-use, and task-specific focus. In contrast to other dermatological systems, such as large-scale vision-language or multimodal systems, the proposed model has been optimized only for dermoscopic lesion classification. The method uses a self-supervised pre-training approach to learn transferable visual representations from dermatological clinical images without using other modalities or proprietary data. While pre-training is on a smaller scale than that of general-purpose vision foundation models, the domain-specific nature of this data enables efficient representation learning tailored to dermoscopy image characteristics.

To differentiate circuit models from segmentation-oriented foundation models, the proposed model was explicitly developed for the classification of individual lesions. The proposed model’s hierarchical vision transformer backbone and token aggregation method strike a balance between global context reasoning and efficiency. These characteristics allow re-use of the pre-trained encoder as a classification backbone across all datasets and training methods. As a reusable, expandable foundation model for dermatological image classification, the proposed model is intended to serve as a foundation for future work. Future work may include segmenting or multimodal results; however, this work aims to create a robust classification foundation model with good generalization. This focus on task-specific models sets the proposed model apart from general-purpose foundation models and matches it with real-world application scenarios for automated dermoscopic diagnostic systems.

### 4.10. Interpretability, Explainability, and Clinical Trust

While the predictive performance of AI systems is crucial, clinicians should also consider how interpretable and trustworthy potential AI systems are when deciding to deploy them in dermatology. This study focuses primarily on the learning and generalization capabilities of AI, as defined by the proposed hierarchical transformer architecture, to facilitate interpretations of AI performance after initial evaluation. Multi-head self-attention facilitates this interpretation by showing how much attention the AI system has given different areas of an image relative to others. These self-attentions yield attention maps representing how much the AI system has attended to each area when making diagnostic predictions, and it is possible to analyze these attention weights by hierarchical levels to understand if the AI system’s attention has been focused on localized details, such as texture variations or color of lesions, versus more general and overarching characteristics of the lesions, such as asymmetrical or irregular lesions.

In addition to identifying attention regions contributing to final classification decisions, being able to calculate the importance of individual tokens in a manner similar to G-CAM, or by means of perturbation studies, helps increase trust in AI systems. Techniques for interpreting AI predictions may support clinicians by leveraging medical evidence contained in the patient’s image(s) to confirm diagnoses. These interpretability techniques will be essential for using AI systems to aid medical decision-making—clinicians can rely on and compare AI-generated predictions with their own clinical reasoning. Later in our research efforts, we will evaluate methods for integrating explainable AI into foundation models tailored to dermatology practice, and we will employ a user-centered framework to assess and maximize the clinical reliability and translational benefit of these models.

### 4.11. Discussion

Results from experiments show that the Transformer-Based Foundation Model provides a consistent performance boost across all dermatological benchmarks studied (ISIC 2018, HAM10000, PH2) under both data-rich and data-poor conditions. In within-dataset evaluations, the foundation model outperformed similar contemporary transformer-based systems. This is likely the result of large-scale dermatology-specific self-supervised pre-training combined with hierarchical attention modeling, which provides improved feature representations of global lesion morphology and coarse-to-fine spatial relationships necessary to differentiate visually similar disease subtypes that exhibit color heterogeneity and irregular boundary topology.

At the end of this study, all quantitative results will be displayed as the mean (± standard deviation) across multiple independent training runs to account for variability due to random initialization and stochastic optimization. Notably, the reported accuracy of the proposed method is statistically higher than that of each baseline method across all datasets and evaluation conditions; however, no formal hypothesis testing has been performed to determine whether the difference in accuracy is statistically significant. Thus, the overall reported accuracy gains from the proposed method should be viewed as empirically validated rather than statistically validated. Future studies should and will include statistically valid formal hypothesis testing and uncertainty analyses to better quantify and strengthen the statistical reliability of comparative evaluations.

In cross-dataset generalization experiments, foundation learning paradigms further demonstrate their advantages over traditional models. During training on a single dataset and evaluation on an external cohort, a baseline model was often unable to retain substantial information due to domain shifts, variability in acquisition methods, demographic differences among patients, and annotation inconsistencies. Traditional transformer-based baseline models (DermViT and Swin Transformer), although showing impressive performance on closed-set evaluations, experienced significant performance decreases during transfer across multiple datasets; however, ensemble CNN and multimodal models displayed moderate performance and resilience to variability at the cost of increased computational complexity. In contrast, the Proposed Foundation Model demonstrated superior preservation of target-domain accuracy and area under the ROC (AUC) value across all transfer directions involving ISIC 2018, HAM10000, and PH2. This observation highlights that incorporating dermatology-specific self-supervised learning produces feature manifolds that are less prone to biases across datasets and more resilient to inter-institutional imaging variance, supporting the model’s applicability in real-world, decentralized clinical environments.

The limited-data fine-tuning findings reinforce the strong potential benefits of foundational pretraining in clinical settings where limited, costly labeled annotations are available. In the label-constrained scenario under very severe conditions (i.e., using only 10% to 25% of the total available data), the conventional transformer and CNN-based solutions, including DermViT, Swin Transformer, Deep Ensemble CNN, and SATU-Net, exhibited unstable convergence and increased variance across training runs. In contrast, the foundational model consistently achieved higher accuracy and lower variance than both traditional and CNN-based solutions, corroborating the great potential of building favorable representations of unlabeled data through large-scale representation learning to produce more consistent, stable models via supervised optimization across multiple datasets.

The results of the ablation study further substantiate the importance of specific components within the overall architecture and training of the proposed model. By removing the self-supervised pretraining component, the largest loss was observed across all datasets, underscoring the central role of self-supervised learning in generating transferable dermatological representations. Further to this point, eliminating hierarchical token aggregation or substituting hierarchical token aggregations for single-scale transformations decreased classification accuracy, particularly within complex boundary lesions and heterogeneous pigmentation, highlighting the contributions of hierarchical attention to fusing global and individual features. Additionally, by omitting augmentation from the dataset, a more moderate, yet consistent, trend of generalizing capability reductions across multiple datasets emerged, indicating the secondary, yet still worthwhile, benefits of augmenting datasets to improve their robustness against noise caused by imaging artifacts and illumination differences.

Collectively, all analyses demonstrated that the performance of the proposed model is not due to a single component, but rather to the collaboration between architectural design and learning strategies. On a computational cost basis, comparisons of the cost vs. speed of the proposed model (Foundation) vs. current lightweight CNN architectures, such as LesionNet, showed that the Foundation model generates moderate increases in training and inference costs relative to LesionNet but remains competitive with existing transformer frameworks and multimodal models. In particular, the foundation model reaches full convergence during the supervised fine-tuning process due to the initialization of weights resulting from pretraining, allowing for greater reductions in total time from initial training to deployment completion and providing for a significant advantage in practical settings when the need arises for recurrent recalibration of the model due to changes in the patient populations or newly emergent dermoscopic lesion trends collected from clinical datasets.

## 5. Conclusions

A Transformer-Based Foundation model for skin pathology imaging is developed using extensive dermatological-specific self-supervised pretraining within a hierarchical vision transformer framework to achieve robust, transferable, and annotation-efficient diagnostic performance across highly diverse clinical data. Higher-performing results from standard, in-dataset evaluations and under challenging cross-dataset transfer conditions demonstrate that foundation-level representation learning significantly improves robustness to changes in the clinical domain caused by medical imaging technologies, differences in patient populations, and the effect of annotation protocols, which typically interfere with the successful implementation of dermatological diagnostic systems into clinical practice. Further experiments evaluating the model’s fine-tuning with limited data reinforce the utility of the described model in the clinical setting. Even when trained on a very small proportion of labeled data, the foundation model demonstrates comparable diagnostic accuracy to traditional supervised models and significantly better convergence stability. These results demonstrate that, through self-supervised pretraining on unlabeled dermatological images, the foundation model reduces the need for extensive manual annotation and significantly minimizes the risk of overfitting, a problem typically observed in small-cohort medical datasets. Ablation analysis further elucidates that, through self-supervised initialization and hierarchical multiscale token aggregation, these methodologies primarily improve diagnostic performance, while advanced data augmentation methods also play meaningful secondary roles.

From a deployment perspective, efficiency studies indicate that while the foundation model incurs a modest increase in computational overhead per epoch compared to lightweight CNN architectures, it converges much faster under supervised training than transformer-based models, making it highly competitive in terms of inference latency. This advantageous trade-off will reduce the training-to-deployment timeframes while maintaining high diagnostic reliability, which is critical to enabling the widespread adoption of AI systems in clinical practice. Moreover, high convergence stability across multiple datasets indicates that foundation-level pretraining can effectively reduce the time required to calibrate models to the realities of continual learning and domain change in clinical practice. Future research will focus on using this framework to integrate clinical workflow across modalities, leveraging patient metadata and imaging context; exploring continuous and federal training strategies for decentralized training; and supporting lesion detection and segmentation to improve decision-support capabilities. The aim of such efforts is to develop deployable, reliable, and generalizable AI systems capable of supporting dermatological diagnoses for a variety of clinical workflows.

## Figures and Tables

**Table 1:** Summary of representative deep learning baselines for dermoscopic image classification, grouped by dataset and architectural category.

Method	Architecture Type	Dataset	Accuracy (%)
ISIC 2018 Dataset			
DermViT [24]	Vision Transformer	ISIC 2018	92.6
Swin-Transformer SkinNet [25]	Hierarchical Transformer	ISIC 2018	93.2
Joint Seg-Cls ViT [9]	Segmentation + Transformer	ISIC 2018	93.0
ViT-Seg Classifier [26]	Vision Transformer	ISIC 2018	92.8
CNN–Transformer HybridNet [27]	CNN + Transformer	ISIC 2018	92.9
HAM10000 Dataset			
Deep Ensemble CNN [5]	CNN Ensemble	HAM10000	96.1
Multimodal Fusion Model [28]	Image + Metadata Network	HAM10000	96.5
YOLOv8 Dermatology [29]	One-Stage Detector	HAM10000	94.9
Architecture Optimization DL [30]	CNN/Transformer Hybrid	HAM10000	96.0
Two-Stage Refined Classifier [31]	Dual CNN Pipeline	HAM10000	95.7
PH2 Dataset			
LesionNet [32]	CNN Residual Network	PH2	96.9
SATU-Net [33]	CNN + Self-Attention	PH2	97.1
Dual-Branch DL [34]	Segmentation + CNN	PH2	97.3
Attention-Guided CNN [35]	Spatial Attention CNN	PH2	96.8
Fusion Transformer CNN [36]	Transformer + CNN	PH2	97.0

**Table 2:** Summary of train/validation/test splitting protocol for each dermoscopic dataset. All splits are performed at the patient level using stratified fixed ratios to prevent data leakage.

Dataset	Split Ratio (Train/Val/Test)	Train Samples	Val Samples	Test Samples	Split Repetitions	Leakage Control
ISIC 2018	70/15/15	∼7100	∼1500	∼1500	Single fixed split	Patient-level split
HAM10000	70/15/15	∼7000	∼1500	∼1500	Single fixed split	Patient-level split
PH2	70/15/15	∼140	∼30	∼30	Single fixed split	Patient-level split

**Table 3:** Summary of experimental hyperparameters and implementation settings used for self-supervised pretraining and supervised fine-tuning across all datasets.

Setting	Value
Image Resolution	224×224
Patch Size	16×16
Transformer Backbone Type	Hierarchical Vision Transformer
Embedding Dimension (*D*)	768
Number of Attention Heads (*H*)	12
Number of Transformer Stages (*L*)	4
Pretraining Optimizer	AdamW
Fine-Tuning Optimizer	AdamW
Pretraining Learning Rate	$3\times 10^{-4}$
Fine-Tuning Learning Rate	$1\times 10^{-4}$
Learning Rate Schedule	Cosine annealing
Pretraining Batch Size	256
Fine-Tuning Batch Size	64
Pretraining Epochs	300
Fine-Tuning Epochs	100
Early Stopping Patience	15 epochs
Weight Decay	$1\times 10^{-2}$
Dropout Rate	0.1
Maximum Stochastic Depth Rate	0.2
Data Augmentations	Cropping, flipping, color jittering, brightness shift, affine transforms
Hardware	GPU-based training environment
Evaluation Repetitions	5 independent runs

**Table 4:** In-dataset classification results on ISIC 2018.

Method	Accuracy (%)	Precision (%)	Recall (%)	F1-score (%)	AUC (%)
DermViT [24]	92.64 ± 0.38	90.91 ± 0.44	88.73 ± 0.41	89.81 ± 0.39	96.12 ± 0.27
Swin Transformer [25]	93.18 ± 0.34	91.24 ± 0.40	89.15 ± 0.37	90.18 ± 0.36	96.68 ± 0.25
Joint Seg–Cls ViT [9]	93.02 ± 0.41	91.08 ± 0.48	89.02 ± 0.45	90.03 ± 0.43	96.59 ± 0.29
ViT-Seg Classifier [26]	92.83 ± 0.39	90.63 ± 0.43	88.49 ± 0.40	89.55 ± 0.39	96.37 ± 0.28
CNN–Transformer HybridNet [27]	92.91 ± 0.36	90.77 ± 0.41	88.88 ± 0.39	89.82 ± 0.37	96.44 ± 0.26
Proposed Foundation Model	94.87 ± 0.27	93.18 ± 0.31	91.47 ± 0.28	92.32 ± 0.26	97.95 ± 0.19

**Table 5:** In-dataset classification results on HAM10000.

Method	Accuracy (%)	Precision (%)	Recall (%)	F1-score (%)	AUC (%)
Deep Ensemble CNN [5]	96.12 ± 0.21	95.04 ± 0.29	94.92 ± 0.27	94.98 ± 0.23	98.21 ± 0.14
Multimodal Fusion Model [28]	96.54 ± 0.24	95.63 ± 0.28	95.31 ± 0.26	95.47 ± 0.22	98.36 ± 0.17
YOLOv8 Dermatology [29]	94.87 ± 0.33	93.12 ± 0.36	92.74 ± 0.34	92.93 ± 0.31	97.74 ± 0.23
Optimized Hybrid DL [30]	96.01 ± 0.26	95.07 ± 0.30	94.89 ± 0.28	94.98 ± 0.25	98.18 ± 0.18
Two-Stage Classifier [31]	95.71 ± 0.30	94.42 ± 0.33	94.01 ± 0.31	94.21 ± 0.29	98.02 ± 0.20
Proposed Foundation Model	97.32 ± 0.18	96.54 ± 0.21	96.09 ± 0.20	96.31 ± 0.19	98.91 ± 0.11

**Table 6:** In-dataset classification results on PH2.

Method	Accuracy (%)	Precision (%)	Recall (%)	F1-score (%)	AUC (%)
LesionNet [32]	96.88 ± 0.31	95.94 ± 0.36	95.61 ± 0.34	95.77 ± 0.32	98.42 ± 0.18
SATU-Net [33]	97.14 ± 0.28	96.23 ± 0.31	95.98 ± 0.29	96.10 ± 0.27	98.61 ± 0.16
Dual-Branch DL [34]	97.32 ± 0.24	96.41 ± 0.28	96.15 ± 0.26	96.28 ± 0.25	98.74 ± 0.14
Attention CNN [35]	96.82 ± 0.29	95.88 ± 0.32	95.53 ± 0.31	95.70 ± 0.30	98.37 ± 0.17
CNN–Transformer Fusion [36]	97.02 ± 0.27	96.11 ± 0.30	95.89 ± 0.28	96.00 ± 0.26	98.55 ± 0.15
Proposed Foundation Model	98.17 ± 0.16	97.54 ± 0.19	97.18 ± 0.18	97.36 ± 0.17	99.23 ± 0.09

**Table 7:** Cross-dataset transfer performance with ISIC 2018 as source dataset.

Method	HAM10000 Accuracy (%)	HAM10000 AUC (%)	PH2 Accuracy (%)	PH2 AUC (%)
DermViT [24]	86.32 ± 0.54	93.41 ± 0.37	84.91 ± 0.61	92.88 ± 0.44
Swin Transformer [25]	87.14 ± 0.49	94.02 ± 0.34	85.73 ± 0.56	93.35 ± 0.40
Joint Seg–Cls ViT [9]	86.87 ± 0.51	93.84 ± 0.36	85.41 ± 0.59	93.08 ± 0.42
CNN–Transformer Hybrid [27]	86.55 ± 0.53	93.67 ± 0.39	85.12 ± 0.60	92.95 ± 0.45
ViT-Seg Classifier [26]	86.44 ± 0.52	93.58 ± 0.38	85.06 ± 0.58	92.91 ± 0.43
Proposed Foundation Model	90.73 ± 0.38	96.12 ± 0.22	90.18 ± 0.41	95.87 ± 0.26

**Table 8:** Cross-dataset transfer performance with HAM10000 as source dataset.

Method	ISIC 2018 Accuracy (%)	ISIC 2018 AUC (%)	PH2 Accuracy (%)	PH2 AUC (%)
Deep Ensemble CNN [5]	87.64 ± 0.43	94.52 ± 0.31	86.11 ± 0.48	93.06 ± 0.37
Multimodal Fusion [28]	88.12 ± 0.41	94.89 ± 0.29	86.64 ± 0.45	93.44 ± 0.34
YOLOv8 Dermatology [29]	85.39 ± 0.58	93.21 ± 0.42	84.74 ± 0.61	92.66 ± 0.45
Optimized Hybrid DL [30]	88.03 ± 0.44	94.82 ± 0.30	86.49 ± 0.47	93.39 ± 0.35
Two-Stage Classifier [31]	87.76 ± 0.46	94.61 ± 0.33	86.27 ± 0.49	93.18 ± 0.38
Proposed Foundation Model	91.45 ± 0.32	96.43 ± 0.20	90.61 ± 0.36	96.02 ± 0.24

**Table 9:** Cross-dataset transfer performance with PH2 as source dataset.

Method	ISIC 2018 Accuracy (%)	ISIC 2018 AUC (%)	HAM10000 Accuracy (%)	HAM10000 AUC (%)
LesionNet [32]	82.93 ± 0.66	91.78 ± 0.48	83.41 ± 0.62	92.05 ± 0.46
SATU-Net [33]	83.61 ± 0.63	92.14 ± 0.45	84.02 ± 0.59	92.51 ± 0.42
Dual-Branch DL [34]	84.21 ± 0.61	92.68 ± 0.43	84.77 ± 0.56	92.92 ± 0.40
Attention CNN [35]	83.14 ± 0.65	91.93 ± 0.47	83.68 ± 0.61	92.22 ± 0.44
CNN–Transformer Fusion [36]	83.89 ± 0.62	92.31 ± 0.44	84.26 ± 0.58	92.67 ± 0.41
Proposed Foundation Model	89.94 ± 0.39	95.87 ± 0.26	90.32 ± 0.35	96.18 ± 0.22

**Table 10:** Limited-data fine-tuning results on ISIC 2018.

Method	10% Data Accuracy (%)	25% Data Accuracy (%)	50% Data Accuracy (%)
DermViT [24]	86.11 ± 0.62	89.54 ± 0.48	91.62 ± 0.39
Swin Transformer [25]	86.74 ± 0.59	90.03 ± 0.46	92.03 ± 0.37
Joint Seg–Cls ViT [9]	86.51 ± 0.61	89.87 ± 0.47	91.89 ± 0.38
ViT-Seg Classifier [26]	86.34 ± 0.63	89.63 ± 0.49	91.72 ± 0.41
CNN–Transformer Hybrid [27]	86.42 ± 0.60	89.81 ± 0.45	91.78 ± 0.40
Proposed Foundation Model	90.48 ± 0.34	92.93 ± 0.28	94.15 ± 0.21

**Table 11:** Limited-data fine-tuning results on HAM10000.

Method	10% Data Accuracy (%)	25% Data Accuracy (%)	50% Data Accuracy (%)
Deep Ensemble CNN [5]	91.74 ± 0.44	94.28 ± 0.31	95.41 ± 0.26
Multimodal Fusion Model [28]	92.12 ± 0.41	94.76 ± 0.29	95.87 ± 0.24
YOLOv8 Dermatology [29]	89.86 ± 0.53	92.51 ± 0.36	94.02 ± 0.31
Optimized Hybrid DL [30]	91.63 ± 0.45	94.61 ± 0.30	95.74 ± 0.25
Two-Stage Classifier [31]	91.28 ± 0.48	94.12 ± 0.33	95.36 ± 0.28
Proposed Foundation Model	94.97 ± 0.27	96.42 ± 0.19	96.96 ± 0.16

**Table 12:** Limited-data fine-tuning results on PH2.

Method	10% Data Accuracy (%)	25% Data Accuracy (%)	50% Data Accuracy (%)
LesionNet [32]	90.63 ± 0.61	93.54 ± 0.43	95.18 ± 0.35
SATU-Net [33]	91.02 ± 0.58	93.91 ± 0.41	95.43 ± 0.32
Dual-Branch DL [34]	91.38 ± 0.56	94.21 ± 0.39	95.74 ± 0.30
Attention CNN [35]	90.72 ± 0.59	93.68 ± 0.42	95.37 ± 0.34
CNN–Transformer Fusion [36]	91.19 ± 0.57	94.04 ± 0.40	95.57 ± 0.33
Proposed Foundation Model	95.86 ± 0.31	97.18 ± 0.22	97.84 ± 0.18

**Table 13:** Ablation study results on ISIC 2018.

Configuration	Accuracy (%)	Precision (%)	Recall (%)	F1-Score (%)	AUC (%)
Without self-supervised pretraining	90.73 ± 0.44	88.32 ± 0.48	86.35 ± 0.46	87.32 ± 0.45	94.68 ± 0.31
Single-scale transformer backbone	91.82 ± 0.41	89.76 ± 0.45	87.91 ± 0.43	88.82 ± 0.42	95.41 ± 0.29
Without token merging	91.37 ± 0.43	89.21 ± 0.47	87.48 ± 0.44	88.33 ± 0.43	95.16 ± 0.30
Without advanced augmentation	92.04 ± 0.40	90.48 ± 0.44	88.61 ± 0.42	89.54 ± 0.41	95.74 ± 0.28
Complete foundation model	94.87 ± 0.27	93.18 ± 0.31	91.47 ± 0.28	92.32 ± 0.26	97.95 ± 0.19

**Table 14:** Ablation study results on HAM10000.

Configuration	Accuracy (%)	Precision (%)	Recall (%)	F1-Score (%)	AUC (%)
Without self-supervised pretraining	94.48 ± 0.31	93.34 ± 0.37	92.98 ± 0.35	93.16 ± 0.33	97.41 ± 0.19
Single-scale transformer backbone	95.42 ± 0.28	94.20 ± 0.33	93.88 ± 0.31	94.04 ± 0.30	97.91 ± 0.18
Without token merging	95.07 ± 0.30	93.92 ± 0.35	93.61 ± 0.33	93.77 ± 0.31	97.72 ± 0.19
Without advanced augmentation	95.71 ± 0.27	94.58 ± 0.32	94.16 ± 0.30	94.36 ± 0.28	98.02 ± 0.18
Complete foundation model	97.32 ± 0.18	96.54 ± 0.21	96.09 ± 0.20	96.31 ± 0.19	98.91 ± 0.11

**Table 15:** Ablation study results on PH2.

Configuration	Accuracy (%)	Precision (%)	Recall (%)	F1-Score (%)	AUC (%)
Without self-supervised pretraining	95.02 ± 0.39	94.17 ± 0.45	93.86 ± 0.42	94.01 ± 0.40	97.51 ± 0.22
Single-scale transformer backbone	96.01 ± 0.34	95.14 ± 0.39	94.81 ± 0.37	94.97 ± 0.35	97.98 ± 0.20
Without token merging	95.67 ± 0.36	94.86 ± 0.41	94.52 ± 0.39	94.69 ± 0.37	97.79 ± 0.21
Without advanced augmentation	96.19 ± 0.32	95.37 ± 0.37	95.02 ± 0.35	95.19 ± 0.33	98.08 ± 0.19
Complete foundation model	98.17 ± 0.16	97.54 ± 0.19	97.18 ± 0.18	97.36 ± 0.17	99.23 ± 0.09

**Table 16:** Computational efficiency and convergence metrics across datasets.

Dataset	Method	Time/Epoch (s)	Convergence Epochs	Inference Latency (ms)
ISIC 2018	LesionNet [32]	41.7 ± 2.1	68 ± 5	3.1 ± 0.2
	DermViT [24]	87.3 ± 3.4	54 ± 4	7.4 ± 0.3
	Swin Transformer [25]	92.6 ± 3.7	56 ± 4	7.9 ± 0.3
	Deep Ensemble CNN [5]	75.1 ± 2.8	60 ± 5	6.2 ± 0.3
	Multimodal Fusion [28]	88.5 ± 3.2	58 ± 4	8.5 ± 0.3
	Proposed Foundation Model	95.8 ± 3.0	42 ± 3	8.3 ± 0.3
HAM10000	LesionNet [32]	43.5 ± 2.0	65 ± 6	3.3 ± 0.2
	DermViT [24]	90.8 ± 3.5	52 ± 4	7.7 ± 0.3
	Swin Transformer [25]	95.1 ± 3.8	55 ± 4	8.1 ± 0.3
	Deep Ensemble CNN [5]	82.3 ± 3.1	58 ± 5	6.5 ± 0.3
	Multimodal Fusion [28]	91.9 ± 3.5	55 ± 5	8.7 ± 0.3
	Proposed Foundation Model	98.6 ± 3.2	40 ± 3	8.5 ± 0.3
PH2	LesionNet [32]	12.6 ± 0.6	58 ± 5	3.0 ± 0.2
	DermViT [24]	30.4 ± 1.5	45 ± 4	7.2 ± 0.3
	Swin Transformer [25]	32.1 ± 1.6	46 ± 4	7.6 ± 0.3
	SATU-Net [33]	26.3 ± 1.3	50 ± 5	6.1 ± 0.3
	Dual-Branch DL [34]	28.9 ± 1.4	48 ± 4	6.4 ± 0.3
	Proposed Foundation Model	33.5 ± 1.6	34 ± 3	8.0 ± 0.3

## Data Availability

The implementation of this work can be found at https://github.com/imashoodnasir/Transformer-Based-Foundation-Learning-for-Skin-Disease-Imaging (accessed on 16 November 2025).
